# Prevention and Mitigation of Frailty Syndrome in Institutionalised Older Adults Through Physical Activity: A Systematic Review

**DOI:** 10.3390/healthcare13030276

**Published:** 2025-01-30

**Authors:** Guillermo Francisco Martínez-Montas, Manuel Sanz-Matesanz, Juan de Dios Benítez-Sillero, Luis Manuel Martínez-Aranda

**Affiliations:** 1 Faculty of Sports Sciences, Department of Sports and Computer Sciences, Universidad Pablo de Olavide, 41013 Seville, Spain; gfmarmon@alu.upo.es; 2 Faculty of Health Sciences, European University Miguel de Cervantes, 47012 Valladolid, Spain; msanzm@uemc.es; 3 Department of Specifics Didactics, Faculty of Education Sciences and Psychology, University of Córdoba, 14071 Cordoba, Spain; 4 Research Group on Sport and Physical Education for Personal and Social Development (GIDESPO), 14071 Cordoba, Spain; 5 Research Group in Sport Psychology for Well-Being and Health, 94100 Enna, Italy; 6 Science-Based Training Research Group (SEJ-680), Physical Performance and Sports Research Center, Universidad Pablo de Olavide, 41013 Seville, Spain

**Keywords:** multicomponent exercise, frailty prevention, functional capacity, exercise interventions, elderly care, health outcomes

## Abstract

**Background/Objectives:** Frailty syndrome significantly impacts the health and quality of life of institutionalised older adults, increasing the risk of adverse outcomes such as disability and mortality. This systematic review aimed to evaluate the effectiveness of physical activity interventions in preventing and mitigating frailty syndrome among institutionalised older adults and to identify key intervention characteristics influencing their effectiveness. **Methods:** A systematic search following PRISMA guidelines was conducted in the Web of Science, PubMed, and Cochrane databases to identify randomised controlled trials published from 2001 to June 2024. Studies involving institutionalised adults aged 60 or older, assessing the impact of physical activity interventions on frailty using validated measures, were included. A narrative synthesis approach was employed to analyse the findings due to the heterogeneity of interventions and settings. **Results:** Twelve randomised controlled trials comprising 1223 participants were included. Multicomponent exercise programmes—combining resistance exercises, balance, and aerobic training—consistently improved frailty indicators, including muscle strength, gait speed, and balance, among others parameters. Frailty reversal occurred in 36% of participants, with interventions showing a reduction in frailty criteria and improved functional autonomy. Programmes integrating physical activity with cognitive or nutritional components demonstrated high efficacy. The control groups showed minimal improvement, highlighting the unique impact of tailored interventions. Despite variability in intervention design, frailty was consistently shown to be reversible in pre-frail and frail individuals, where the benefits were evident including for individuals over 85 years old. **Conclusions:** Physical activity interventions, particularly multicomponent exercise programmes, are effective in reducing frailty and improving health outcomes in institutionalised older adults. Future research should focus on optimising intervention characteristics and exploring the long-term sustainability of benefits in diverse populations. These findings reinforce the importance of exercise as a cornerstone in frailty management.

## 1. Introduction

Frailty is a syndrome influenced by numerous components that play a crucial role in its development, leading to various problems or pathologies such as an increased risk of falls, hospitalisation, disability, or mortality. All these consequences, combined with the confusion between sarcopenia and frailty, as well as the potential intervention methodologies to mitigate their effects—such as physical exercise—necessitate in-depth study.

### 1.1. Frailty Syndrome in Institutionalised Older Adults

Despite medical advancements in preventing and treating age-related health complications, frailty syndrome remains prevalent among older adults. This condition limits daily activities due to sarcopenia and dynapenia, leading to poor health outcomes, reduced functional capacity, fatigue, and falls [1]. Frailty is a biological syndrome characterised by diminished reserves to withstand stressors, resulting from cumulative physiological impairments, and is associated with an increased risk of adverse outcomes, including falls, hospitalisation, disability, and mortality [2,3].

The Spanish Ministry of Health highlights the importance of assessing functional status rather than disease presence when evaluating frailty. The World Health Organization (WHO), in collaboration with the ADVANTAGE Joint Action, defines frailty as “an age-related decline in physiological systems, reducing functional reserve, increasing vulnerability to stressors, and heightening the risk of adverse health outcomes” [4,5]. This definition shifts the focus from disease diagnosis to functionality, now recognised as the best predictor of adverse events such as falls, hospitalisation, disability, and death, regardless of multimorbidity [5,6,7].

Frailty is closely linked to disability and other adverse health outcomes, including depression, cardiovascular events, and increased rates of institutionalisation and mortality [8]. A growing proportion of older adults, often affected by functional dependence, multimorbidity, and polypharmacy, reside in long-term care facilities. These residents typically spend most of their time sitting or lying down and rarely engage in exercise, which exacerbates physical decline [9,10]. Over half of these residents lose the ability to independently perform at least one daily living activity within two years of admission [11]. Institutionalised older adults often exhibit reduced intrinsic capacity, encompassing physical and mental abilities, due to the combined effects of ageing and physical inactivity. This leads to physiological changes such as muscle mass loss, impaired neural mechanisms for strength and power, reduced bone mineral density, and cognitive decline [12,13].

### 1.2. Frailty Syndrome and Sarcopenia

Although sarcopenia and frailty partially overlap due to shared physiological causes, they are distinct in several aspects. Frailty is a more complex syndrome involving multiple biopsychosocial mechanisms, whereas sarcopenia is defined as the loss of muscle mass, strength, and function [14]. Frailty, in contrast, is a state of vulnerability resulting from the deterioration of various physiological systems and is strongly associated with ageing. However, its prevalence varies significantly among older individuals [15]. When considering the physical frailty phenotype, there is notable overlap with sarcopenia, particularly in shared clinical features such as reduced grip strength and slower gait speed [16]. Sarcopenia itself may act as a risk factor for frailty, defined as a physical phenotype. Both syndromes share common pathogenic mechanisms [17] and overlapping diagnostic criteria, including reduced grip strength and gait speed. Together, they are the primary causes of functional decline in older adults, leading to disability, falls, poor quality of life, institutionalisation, and mortality.

While there is a broad consensus on the theoretical framework of frailty, its clinical identification is challenging due to its complex pathophysiology, heterogeneous phenotypic manifestations, intraindividual variability in severity, and the existence of multiple operational definitions [18]. It is important to highlight that frailty is not an inevitable consequence of ageing. Sarcopenia, malnutrition, reduced physical activity, chronic diseases, and polypharmacy all contribute to frailty but are modifiable with appropriate interventions [19,20].

### 1.3. Frailty and Falls

Falls are a leading cause of hospitalisation among frail older adults [21,22]. While most fall-related injuries are minor—such as bruises, lacerations, sprains, and strains—some can result in severe, long-term consequences. Serious injuries include joint dislocations, fractures, and concussions, with fractures being particularly significant as they account for a substantial proportion of morbidity and mortality among older adults [23]. Falls and their associated consequences are recognised as a global health concern affecting the ageing population. Approximately 35% of community-dwelling individuals aged 65 or older experience at least one fall annually, a figure that rises to 50% among those aged 80 or older [24]. Among institutionalised older adults, the situation is even more concerning, as falls may either precede or result from admission to care homes. In this population, the fall rate increases to 50%, with 12% to 40% experiencing recurrent falls [25].

The aetiology of falls is considered multifactorial, resulting from complex interactions between intrinsic and extrinsic risk factors [26]. Extrinsic factors include environmental risks, such as slippery floors, poorly designed stairs, and inadequate lighting, as well as socioeconomic challenges like low income, limited education, and social isolation. Intrinsic factors are often linked to physical capabilities, including muscle mass loss, reduced strength and function (sarcopenia), impaired balance, and diminished mobility. In this regard, exercise programmes have proven effective in reducing fall rates among older adults in care homes. These programmes typically include aerobic and strength-based exercises, providing at least three hours of physical activity per week [27]. Such interventions have shown positive effects on both mobility and physical functioning, addressing key risk factors for falls and improving overall quality of life.

### 1.4. Physical Activity and Its Effectiveness on Frailty

As previously noted, frailty is not a static condition but a continuous cycle. Individuals can transition from being healthy to pre-frail, frail, and eventually disabled, though early interventions can reverse this process to some extent [28]. Early identification and intervention are critical for improving the prognosis of frailty.

Physical activity is considered the most cost-effective primary intervention to delay and reverse frailty [29]. Exercise-based programmes can preserve and enhance muscle mass, strength, and power in institutionalised older adults, improving their quality of life, functionality, and independence [30,31]. Recent clinical guidelines strongly recommend that frail older adults participate in supervised, progressive exercise programmes that include resistance, balance, and aerobic training [32,33]. These interventions have been shown to effectively delay and reduce frailty in institutionalised settings, improving participants’ functional capacity and health-related quality of life [34]. Aerobic resistance training, according to [35], can improve maximum oxygen consumption by 10% to 15%. Resistance training is particularly effective for increasing muscle strength and mass. While muscle mass gains may be limited in frail older adults, resistance training can improve muscle strength significantly, by approximately 110% in institutionalised patients.

Similarly, the United States Department of Health and Human Services [36] recommends multicomponent exercises, including balance training alongside aerobic and muscle-strengthening activities, to maintain health in older adults. Multicomponent exercises have been reported to enhance physical performance, which is essential for mobility, independence, and managing chronic disease burdens [32,33,37].

The International Association of Gerontology and Geriatrics—Global Aging Research Network (IAGG-GARN) and the Clinical Section of the European Region of the IAGG also recommend multicomponent exercise programmes, combining balance, muscle strengthening, and aerobic training at moderate intensity. These have proven effective in enhancing the performance of the activities of daily living (ADLs) in older adults residing in long-term care facilities [32]. Moreover, research on frailty interventions has highlighted the role of dietary and exercise interventions, such as anti-inflammatory diets, which can counteract the adverse effects of frailty [38].

To date, systematic reviews focusing on institutionalised patients with frailty and the potential effects of physical exercise on them are non-existent, despite their significance and considerable representation in society. As previously described, frail patients are predominantly admitted to specialised care facilities. Such admission entails lifestyle changes that must be studied in detail. Thus far, systematic reviews have centred on the application of specific modalities of physical exercise or interventions and their outcomes in patients [39,40], or on a general analysis of the frail population, examining the effects of exercise and the cost–benefit considerations involved [41,42]. However, it is essential to specifically analyse institutionalised patients and observe the unique aspects they present.

Despite its proven benefits, physical activity levels among institutionalised older adults remain low [43]. Factors such as the dependency-inducing environments of care facilities and the vulnerable characteristics of residents often exclude this group from health promotion programmes [44,45]. Moreover, the identification of cost-effective interventions to prevent frailty is a critical public health challenge. For all these reasons, the main aim of this review was to assess the effectiveness of physical activity interventions in preventing and mitigating frailty syndrome among institutionalised older adults. Additionally, the review examined potential moderating effects, including intervention characteristics (e.g., type of exercise, programme duration, and training volume) and participant characteristics (e.g., setting, functional status, and cognitive status). The questions this review aims to address are based on the following: determining the effectiveness of interventions to prevent or reduce frailty in older adults residing in care homes or nursing facilities; examining whether physical activity reduces disability and adverse events in adults over 60 with any component of frailty syndrome; and assessing whether physical activity is the most suitable method to treat frailty and identifying the optimal timing for its implementation, with the goal of preventing or mitigating its effects.

## 2. Materials and Methods

### 2.1. Experimental Design

The review was conducted in compliance with the PRISMA guidelines (Preferred Reporting Items for Systematic Reviews and Meta-Analyses) [46]. To identify the studies in the first instance, two of the authors (G.F.M.-M. and J.d.D.B.-S.) conducted independent searches in the electronic databases Web of Science (WoS), MEDLINE (PubMed), and the Cochrane Central Register of Controlled Trials (Cochrane), using specific keywords and medical subject headings (MeSH) to identify randomised controlled trials.

A methodological search filter was applied to include only results published from 2001 onwards (until June 2024), as this marks the publication year of the current criteria defining frailty [19] which are fundamental to the development of this research. A narrative synthesis approach was employed to examine the results.

Descriptors were organised as follows, converted into controlled language, and combined using the Boolean operators “AND” and “OR” with inferred or related terms:

Part 1 (Ageing): aging, older adult, geriatric patient, elderly;

Part 2 (Frailty): frailty, pre-frail, frailty syndrome, frail elderly;

Part 3 (Physical Activity): physical activity, exercise, intervention;

Part 4 (Institutionalisation): institutionalised, resident, internal, nursing homes.

### 2.2. Study Criteria

For study selection, we followed the principles of evidence-based medicine, using the PICOS criteria [47] as a framework: participants or problem, intervention, comparison, outcomes, and study design.

The inclusion criteria were as follows:

(a) Studies involving institutionalised adults aged 60 or older (population);
(b) Studies assessing the effects of physical exercise interventions or levels of physical activity (intervention);
(c) Studies including a control group or groups with different training loads (comparison);
(d) Studies evaluating pre-frailty, frailty, or sarcopenia (outcome);
(e) Original articles: randomised controlled trials published in Spanish or English (study design).

Articles were excluded if participants had a specific condition that could affect motor capacity or functionality (e.g., Parkinson’s disease, multiple sclerosis, or cerebrovascular accidents), including COVID-19 confinement, or if the study focused solely on patients with a terminal diagnosis. Additionally, the following were excluded: narrative or systematic reviews, with or without meta-analysis; observational studies; grey literature; conference proceedings; or non-peer-reviewed publications. Studies that did not evaluate frailty syndrome, relied exclusively on education-based interventions for an active lifestyle or alternative therapies, or were unrelated to institutions, care homes, or residences for older adults were also excluded.

**Figure 1:**
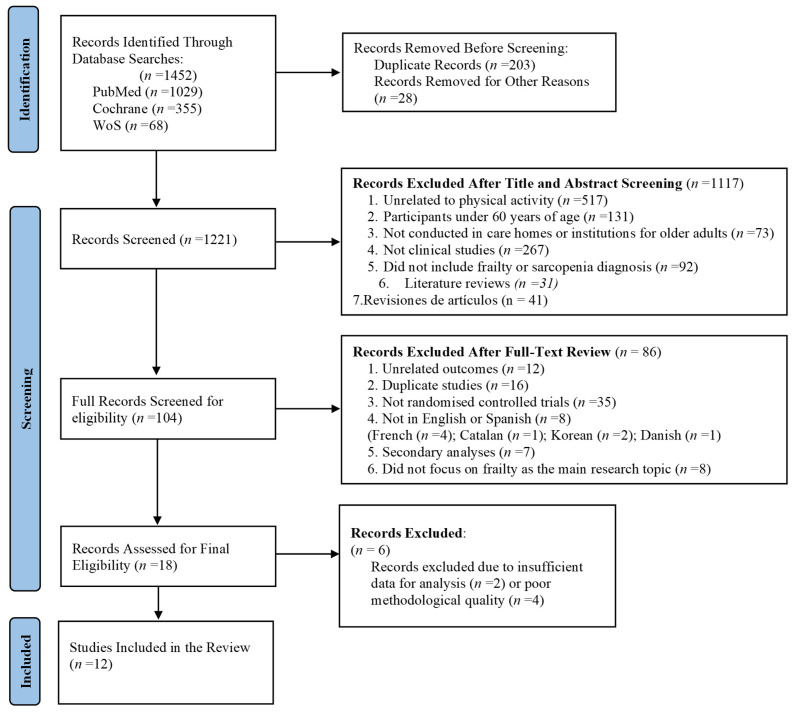
Flow diagram of the study selection and inclusion process [46].

Finally, all the articles included in the review, as well as those cited in the Introduction and Discussion, were reviewed in order to delve deeper into the topic and identify potential new references. However, these studies did not meet the inclusion criteria for the review and/or fell outside the scope. The reason for the decision not to include them in the flowchart (Figure 1) is that they were excluded from the original search process in the databases, although the consultation was conducted.

### 2.3. Variables of Interest

The primary outcome of interest was frailty, as assessed by any validated scale, measurement, or index. Examples include the frailty index, Fried’s frailty criteria based on the phenotypic model [19], the study of osteoporotic fractures (SOF) index [48], the Tilburg frailty indicator [49], or sarcopenia identified using the diagnostic algorithm of the Foundation for the National Institutes of Health [50].

Secondary outcomes included the degree of change, or lack thereof, through physical activity, measured using any validated scale, measurement, or index in the following domains:

- Cognition (e.g., assessed using the mini-mental state examination).
- Quality of life (e.g., assessed through self-reported measures like EuroQol).
- Activities of daily living (ADLs) (e.g., assessed using the Barthel index [51], Katz index, or others).
- Functional capacity (e.g., assessed using the physical activity scale for the elderly).
- Depression and other mental health outcomes (e.g., assessed using the geriatric depression scale—Yesavage).

These outcomes also included the changes, or lack thereof, in analytical parameters (e.g., measured through clinical tests) and the prevalence of adverse outcomes such as falls, fractures, mortality, hospitalisation, and comorbidities (e.g., indicated by medical records or self-reported data).

### 2.4. Data Extraction and Synthesis

The reviewers G.F.M.-M. and L.M.M.-A. independently used the search terms to examine the scientific literature through the different selected metasearch engines and evaluate the titles and abstracts. After excluding irrelevant studies, the full texts were reviewed to identify the eligible literature. Extracted data included the following: (1) basic information (author names, year of publication, location, and study methods); (2) participant details (characteristics, sample size, mean age, and sex); and (3) intervention characteristics (format, frequency, intensity, and total intervention duration).

Differences in the populations, interventions, comparators, and outcomes across the included studies focusing on the clinical/medical component of this review prevented direct comparisons, making a meta-analysis unfeasible. Consequently, the results of these studies were synthesised narratively and presented in tabular form. In the event of any discrepancies during the inclusion process, these were discussed and resolved by consensus. If such discrepancies persisted, a third reviewer went on to moderate the final consensus process in the inclusion of the article (J.d.D.B.-S.).

### 2.5. Quality Assessment

The methodological quality of each study was assessed using the PEDro scale (Physiotherapy Evidence Database) [52]. The evaluation was based on the information reported in each study. In cases of uncertainty or missing data, the criterion was marked as not meeting the PEDro scale recommendations.

At the conclusion of the search process, each selected article was independently assessed before retrieval to determine its methodological validity for inclusion in this systematic review. To ensure the quality of the evidence analysed, a cutoff point was applied for the inclusion of studies focused on the clinical/medical component. Experimental studies were considered to meet a minimum quality threshold if they scored at least five “Yes” ratings on the PEDro scale checklist (PEDro scores ≥ 5).

The methodological quality of the studies is summarised in Table 1. A total of 18 studies were independently evaluated for final eligibility. The final PEDro score was calculated as the sum of criteria rated as satisfactory among Criteria 2 to 11. Criterion 1, which evaluates the external validity of the study, was not included in the final score. The PEDro scores ranged from 6 to 9 out of a maximum of 10 points for 12 studies, with a mean and standard deviation of 7.5 ± 1 points. All selected studies met Criteria 10 and 11.

Based on the final PEDro scale scores, twelve studies were included for additional analysis, while four studies were excluded for failing to meet the minimum requirement of five “Yes” responses on the critical appraisal checklist. Additionally, two other studies were excluded as they did not contain sufficient valid results for analysis.

All included studies were randomised controlled trials (RCTs). None of the studies achieved the maximum score of 10 “Yes” responses, with the highest score of 9 being obtained by only two studies [53,62] (see Table 1). The most frequently identified methodological weakness was related to blinding the therapists administering the therapy or exercises. In 11 studies, therapists were not blinded to treatment allocation (P6), and only 1 study [56] implemented therapist blinding. Due to the nature of the interventions, practical challenges in blinding the individuals delivering the exercises were acknowledged. Four studies [55,56,59,60] did not fulfilled the blinding for all subjects (P5). 

In three studies [33,54,56], allocation concealment was not implemented (P3). However, all studies used random allocation (P2), and the groups were similar at baseline (P4) in six studies [53,55,57,58,61,62].

Regarding assessor blinding (P7), one study lacked clarity on this criterion [58]. In two studies [54,56], key outcome measures were not obtained for more than 85% of the initially allocated participants (P8). Additionally, this criterion was unclear in two other studies [57,61].

In one study [58], not all participants with available outcome measures received the assigned treatment or control condition as allocated (P9). Conversely, all included studies reported key outcomes (P10) and provided point estimates and measures of variability (P11).

## 3. Results

### 3.1. Selection of Studies

The references retrieved from each database were exported to Refworks, resulting in a total of 1452 potentially relevant references. After removing duplicates (*n* = 203) and other exclusions (*n* = 28), 1221 documents were analysed by title and abstract during the initial screening phase. Following this preliminary review, 1117 references were excluded for not meeting the PICOS criteria [47] and the inclusion criteria described earlier. A total of 104 records passed the first screening phase and underwent full-text review. Subsequently, 86 references were excluded for the following reasons: unrelated outcomes (*n* = 12), duplicate studies (*n* = 16), not randomised controlled trials (*n* = 35), language restrictions (French = 4, Catalan = 1, Korean = 2, Danish = 1), secondary analyses (*n* = 7), and studies not focusing on frailty as the main research topic (*n* = 8). The methodological quality of the remaining 18 studies was assessed. After the final eligibility review (*n* = 18), six records were excluded due to insufficient data for analysis (*n* = 2) or poor methodological quality (*n* = 4). A PRISMA flow diagram [46] was used to illustrate the detailed study selection process, as shown in Figure 1.

### 3.2. Study Characteristics

The publication dates of the 12 included studies ranged from 2018 to 2024, and all were published in English. The following sections summarise the main characteristics of the included studies.

Five of the included studies were conducted in Spain [34,54,55,58,61]. Of the remaining seven studies, four were conducted in Asia: one in China [53], one in Japan [57], one in Singapore [33], and one in Indonesia [62]. Two studies were conducted in Brazil [59,60], and one study was conducted in New Zealand [56] (see Table 2).

Participants were recruited from nursing homes or long-term care institutions in 10 studies [34,53,54,55,56,58,59,60,61,62]. One study recruited participants from community senior care centres [33], and another from a rehabilitation centre for older adults [57].

The 12 studies analysed in this review included a total of 1223 older adults. The number of participants per study ranged from 24 [55] (personalised multicomponent exercise), to 520 [56] (physical activity programme in care homes). Two studies [61,62] utilised the same sample (*n* = 34).

Regarding the age of the participants, three studies included older adults aged 60 years or above [59,60,62]. In three other studies, the inclusion criterion was 65 years or older [33,56,57]. In the remaining studies, participants were aged 70 years or older [34,53,54,55,58,61] (Table 2).

Regarding the frailty condition of the participants, seven studies required frailty or pre-frailty as a mandatory inclusion criterion [33,53,56,58,59,60,62].

In two studies [34,54], the level of frailty was measured using the Barthel index [51], with a score of ≥50 points as an inclusion criterion. In addition, López-López et al. [61] considered both frailty using the Barthel index and sarcopenia as inclusion criteria. Another study [57] included older adults with at least one frailty symptom according to Fried’s criteria [19]. Finally, one study [55] used sarcopenia alone as the inclusion criterion to classify participants as frail.

### 3.3. Definition of Frailty

Over time, several operational definitions of frailty have been proposed, primarily inspired by two models: the frailty phenotype by Fried et al. [19] and the frailty index by Rockwood et al. [15]. The phenotypic model of frailty is based on five components: (1) unintentional weight loss, (2) muscle weakness, (3) exhaustion, (4) slow walking speed, and (5) low physical activity [18]. For Mitnitski et al. [63], the frailty index is grounded in the cumulative deficit paradigm and includes health deficits spanning multiple domains. As such, the frailty index can be viewed as an approximate measure of ageing. The studies included in this review utilised different definitions of frailty (see Table 3).

The most frequently cited definition of frailty, referenced in six studies [34,53,54,55,58,59], describes frailty as a reduction in physiological reserve and multisystem dysfunction in older adults, leading to increased vulnerability and a higher risk of adverse outcomes.

Three other studies [56,57,61] incorporate fall risk and disability into their definitions of frailty. Additionally, one study [62] adopts the above definition while also considering fall risk and disability as part of a broader concept of frailty in older adults.

Regarding tools for assessing and measuring frailty, eight studies [33,34,53,54,57,58,59,62] used Fried et al.’s frailty phenotype (FFP) [19]. Four studies [34,53,57,61] incorporated broader parameters, including quality of life and activities of daily living (ADLs), into their frailty assessments.

Cognitive function was included as a parameter in three studies [53,54,56]. Depression was considered in one study [59], which identified frailty through a combination of biomedical, functional, and psychosocial indicators.

Some studies [54,55,56,60,61] focused on functional capacity and fall risk as key parameters, using tools like the timed up-and-go test (TUG), which assesses dynamic balance and functional mobility in older adults [71], and the Berg balance scale (BBS), developed by Katherine Berg in 1989 to evaluate balance capacity in older adults, with an initial target population averaging 73 years of age [69].

### 3.4. Frailty Phenotype (FP)

Eleven of the studies included in this review utilised Fried et al.’s frailty phenotype [19], which provides a standardised, physiologically-based definition applicable to the spectrum of frailty presentations observed in older adults. Its clear criteria are relatively simple and cost-effective to implement, offering a foundation for standardised frailty screening and risk assessment in older populations. This approach can potentially be used to estimate the clinical risk of adverse outcomes. Fried’s frailty phenotype index commonly involves five criteria: unintentional weight loss, weakness, low energy expenditure, slow walking speed, and weak grip strength (see Table 4).

The functionality of frailty indicators varied across studies. In six studies [33,34,53,54,59,62], muscle strength was measured using isometric knee extension in the dominant leg with the participant seated. Another study [57] assessed muscle strength through maximum grip strength, measured in kilograms using a portable dynamometer. Two studies [60,61] did not provide detailed information.

Two studies [34,54] that utilised Fried’s frailty phenotype [19] also employed the SOF Index (study of osteoporotic fractures) [48] and the Tilburg frailty indicator, which includes 15 variables assessing physical, cognitive, and social aspects [49].

### 3.5. Characteristics of the Interventions

The interventions examined in the included studies were primarily grouped into three categories: routine resistance and strength exercises (*n* = 4) [33,53,57,60], multicomponent exercises (*n* = 4) [34,54,59,61], and specific exercise programmes (*n* = 4) [33,55,58,62].

The control group conditions varied across studies. In four studies [53,58,59,60], participants in the control group continued their usual activities without any intervention. One study [54] included cognitive exercises and dual-task training in the control group. In several studies [34,61,62], the control group performed routine daily activities organised by care staff, carried out physical activities without resistance or progression [56], or received usual care with education [57]. One study [33] incorporated a placebo nutritional intervention in the control group, while another study [55] included two experimental groups: one with a short-term intervention (4 weeks) and the other one with a long-term intervention (24 weeks).

The duration of interventions varied, with 12 weeks being the most common, observed in three studies [59,60,61]. The longest intervention lasted 12 months [53,56], while the shortest was 4 weeks [55,62]. Further details on the interventions and their characteristics are provided in Table 5.

A comprehensive analysis of 12 studies demonstrates the substantial impact of various exercise interventions on frailty and associated health outcomes in older adults. Multicomponent and tailored exercise programmes consistently improved physical performance, mobility, and quality of life while reducing frailty prevalence. Integrated exercise interventions, such as those combining strength, balance, and cognitive training, led to significant gains in SPPB scores, walking speed, and gait efficiency [53,54,61]. These benefits extended to reductions in anxiety and frailty-related parameters, including cross-sectional muscle area and fall incidence [34,54].

Specific interventions, such as the Vivifrail and Otago programs, showed marked improvements in balance (BBS), fall efficacy, SPPB, and functional mobility, such as TUG test, with large effect sizes and no adverse effects reported [54,58,62]. In this context, studies such as that by Courel-Ibáñez et al. [55] demonstrate that frailty was reversed in 36% of participants, while 59% achieved a state of high functional autonomy, maintaining it even after detraining. Conversely, 83% of participants who reached a pre-frail state were unable to maintain their autonomy following detraining.

Programmes combining resistance training with physical activity (RPA) or multiple-component exercises programmes, achieved notable reductions in frailty scores, particularly in mobility-related outcomes such as the TUG test, BBS, grip strength, and walking speed [57,59,60]. Nutritional supplementation, when integrated with physical interventions, further enhanced outcomes, such as muscle mass preservation and functional improvements, as shown in the OEP+N programme [33,58].

Significant reductions in frailty prevalence were observed in all groups, including the CG (15%), but were significantly higher (35.6% to 47.8%) across multicomponent interventions, with the odds of reversing frailty being highest in combined interventions (odds ratio [OR] 5.00), followed by physical (OR 4.05), nutritional (OR 2.98), and cognitive interventions (OR 2.89) [33]. These effects were sustained over 12 months, with adherence rates between 79 and 94% depending on the different study groups. Programmes such as S-UpR preserved step counts better than the control groups over extended periods, reinforcing their feasibility and acceptability [56].

However, despite the effectiveness of the interventions, it is important to highlight data regarding dropout rates, which range from 0% to 44.7%, with the highest rate being associated with nutritional intolerances [58]. Similarly, the general dropout rate falls between 7% and 20%, with studies citing reasons such as relocation to another care facility [33,56,62], loss of interest [53,55,57,59], severe hospitalisations [33,56,57], or deaths [33,53,56].

Regarding the adverse effects of the interventions, 9 out of 12 studies reported no adverse effects of any kind. The remaining three studies noted issues such as falls [56], joint pain [33], and problems with food intolerance [58].

Overall, these findings highlight the efficacy of exercise-based and multidisciplinary approaches in reducing frailty and enhancing functional independence in older adults, with improvements persisting beyond the intervention period [61,62].

Table 6 provides an overview of the analysis of the results from interventions conducted in the included studies on the variable of frailty, measured using any validated scale, metric, or index, or assessed through a limited set of indicators.

## 4. Discussion

This review aimed to examine the scope and safety of physical activity in addressing frailty among institutionalised older adults, with the additional objective of determining whether sufficient information exists to answer questions regarding the impact of preventing frailty levels through physical activity interventions and which factors influence the effectiveness of these interventions.

Although there are numerous interventions for frail older adults, few studies specifically address frailty in institutionalised settings. Many studies were excluded due to the lack of validated pre- and post-intervention frailty measures, highlighting the early developmental stage of this field, dominated by observational studies rather than well-designed randomised controlled trials. This review identified 12 randomised controlled trials meeting the inclusion criteria. The heterogeneity of settings and interventions precluded a meta-analysis, but the variety provided valuable insights into the types of interventions most effective in preventing, delaying, or reversing frailty.

Not all studies analysed in this review focused solely on muscle-strengthening interventions. Many combined programmes addressed the activities of daily living, walking, balance, nutritional supplementation, and other components, potentially enhancing the overall benefits of physical activity in improving frailty.

The studies reported a wide range of ages and gender distributions and included individuals at various stages of frailty risk, including pre-frail, frail, and sarcopenic individuals. In terms of age, interventions demonstrated effectiveness across all age groups, with physical exercise programmes proving beneficial for participants aged 60 and older. As an example, four studies focusing on “older” institutionalised participants with a mean age >85 years [34,56,58,61] showed significant improvements in physical frailty components such as gait speed, grip strength, and aerobic endurance.

Research on healthy older adults highlights that physical exercise improves cognitive abilities and quality of life [33,53,58], with regular participation in sports being a key factor influencing this improvement [75]. Frailty, on the other hand, is consistently associated with a decline in quality of life, regardless of the measures used, and this negative impact worsens as frailty progresses [56,59]. Another notable finding in experimental group participants is that while frailty and fall risk were significant predictors at 15% [60], functional capacity was a significant predictor of autonomy in activities of daily living (ADLs), accounting for 22%. Additionally, most variables showed significant correlations, except for the Barthel index scores [61] and 6 min walk test (6MWT) values [54,57].

Previous studies have shown the general benefits of physical exercise for older adults in residential care settings, in order to improve their functional capacity, autonomy, and fall risk, all related to frailty. However, although there is ongoing debate about the most effective type of exercise [76,77,78], group-based interventions have proven more successful than individually conducted sessions [79]. In this context, two studies specifically evaluating the effectiveness of exercise interventions on frailty [59,61] concluded that these programmes were beneficial for various physical performance outcomes. However, neither study separately analysed the results of group-based versus individual interventions.

Multicomponent exercise programmes [34,54,55,59,61,80] have consistently demonstrated improvements in physical performance, particularly muscle strength, balance, and endurance, in frail older adults. Notably, balance plays a key role in enhancing physical function and activity levels [81]. Evidence suggests that these programs, which combine resistance, strength, and balance training, are highly effective in improving gait and overall physical performance, especially in frail populations [82,83,84,85].

Institutionalised older adults with sarcopenia benefit significantly from personalised exercise programmes like “Vivifrail,” both in the short term (4 weeks) and long term (24 weeks). Although functional fitness declined by 10–25% after detraining periods of 6 and 14 weeks, it remained higher than baseline levels [55]. This protective effect of physical exercise has been previously confirmed in community-dwelling older adults [86,87] and more recently observed in institutionalised populations [55,56,58,80].

The statistically significant improvements in strength observed in some studies may be attributed to the moderate-to-high intensity of multicomponent training, which was progressively increased monthly. Recent research further supports a strong link between high-intensity training and enhanced strength [35,77,88]. Progressive resistance training has been shown to produce greater increases in lean mass and muscle strength compared to aerobic exercises performed at home, particularly in frail men and women [35,89,90,91]. These improvements translate into better functional performance and reduced self-reported disability [18]. Depending on the type of exercise and gender, 1RM strength increases by 17–43% from baseline [92]. A critical factor in resistance training programmes is the intensity or the amount of weight lifted.

Contradictory findings in lower-limb strength training trials could potentially be addressed by incorporating strength training into more comprehensive multicomponent exercise programs. In this review, trials involving multicomponent programmes focused on resistance/strength, balance, flexibility exercises, and aerobic endurance approach as well [34,54,59,61]. All these trials reported statistically significant improvements in falls, mobility, balance, functional capacity, muscle strength, and body composition. Fear of falling was significantly reduced following multicomponent exercise, except in one trial where no significant changes were observed due to early termination of the intervention in over a third of participants caused by COVID-19 disruptions [56].

Studies reporting significant gains in muscle strength [93], reductions in frailty parameters [34], and improvements in functionality and balance [94] often included strength training, either alone or combined with aerobic exercises, with the latter yielding the best results [35,95]. Research also indicates that frail older adults retain the ability to adapt to moderate-to-high intensity or power training [96]. Moderate-intensity exercise has been shown to benefit functional parameters [97,98], reduce fall risk [99], and improve balance [100] and self-reported health. However, higher-intensity programmes appear to produce even greater results [35,89]. Beyond intensity, factors such as the duration, method, and type of exercise also likely influence outcomes in studies involving exercise programmes for frail older adults. Considering these findings, it is important to highlight that other studies combining multicomponent exercises with cognitive training and nutritional supplementation have reported superior outcomes [53,54,56,57,59].

Some studies have shown mixed results [97,98,99], where multidomain cardiovascular interventions involving participants aged 70–78 years (*n* = 3526) [101] and the Alzheimer’s Multidomain Prevention Trial (MAPT), which combined polyunsaturated fatty acids, cognitive training, and physical activity for participants aged 70 and older (*n* = 1689) [102], did not yield significant effects on cognitive decline. However, the Finnish Geriatric Intervention Study to Prevent Cognitive Impairment and Disability (FINGER) demonstrated significant efficacy in delaying cognitive deterioration [103].

Furthermore, regarding other variables identified in some studies included in this review [33,59], improvements in biochemical and functional capacity (strength, speed, and agility) were observed among residents of long-term care institutions following a training programme. In this context, Sadjapong et al. [92] showed that while improvements in biochemical, anthropometric, or inflammatory variables were not evident when comparing the final raw values between EG and CG, significant enhancements in serum glucose, insulin, total cholesterol, triglycerides, vitamin D3, and CRP levels were noted when comparing the pre–post-intervention stages within the EG.

The positive effects of physical exercise-based interventions, which become more effective when combined with cognitive training protocols and nutritional support, provide short- to medium-term evidence of efficacy. These studies typically span between 4 and 24 weeks, with follow-ups of up to 12 months. However, the long-term benefits remain uncertain due to the absence of specific studies meeting the criteria of this review that track outcomes beyond the intervention period into subsequent years. This represents one of the most common limitations identified in the literature.

Conversely, long-term observational studies analysing the progression of frailty in patients with specific conditions [104] have highlighted a degenerative trend among hospitalised patients, underscoring the importance of maintaining such programmes. These studies could be complemented by long-term intervention or monitoring studies on physical exercise programmes to determine whether the demonstrated persistence and worsening of frailty in this population could be mitigated. Additionally, the studies included in this review emphasise that the benefits of training programmes focused on reducing frailty diminish significantly after periods of detraining [55]. This further underscores the importance of designing long-term programmes and implementing sustainable follow-ups to ensure patient adherence. New research should explore ways to maintain these programmes over time and preserve the benefits they provide to patients.

Furthermore, analysing adherence to training programmes must consider not only their proven effectiveness in older adults but also their feasibility and cost, which are major limitations identified in the studies reviewed. Implementing these programmes in institutional settings poses logistical and economic challenges, such as the availability of trained personnel, adequate infrastructure, and integration into residents’ daily routines. These factors have been examined in other reviews, such as that by Calonge-Pascual et al. [105], which highlights the critical role of social and economic factors in determining the feasibility and long-term adherence to health-related exercise programmes, revealing significant gaps in this area.

Incorporating analyses on how to overcome these barriers could enhance the applicability of the findings, alongside a potential evaluation of the costs associated with falls and frailty-related incidents compared to the funding required for exercise programmes in healthcare institutions.

This review reported important findings; however, several limitations must be acknowledged. First, the search was restricted to articles published in English and Spanish. Excluding studies in other languages may have limited access to potentially significant findings developed in cultural and socioeconomic contexts different from those considered in this review. Second, not all available scientific literature platforms were explored. While the databases used were appropriate, expanding the search to additional platforms could provide a more comprehensive understanding of the topic.

Moreover, many studies were excluded due to the absence of an operational definition of frailty for participant selection and/or the use of varying criteria to evaluate frailty pre- and post-intervention. In this regard, the different definitions of frailty and measurement criteria used in the included studies represent a significant source of heterogeneity. For instance, while some studies applied Fried’s frailty phenotype, others utilised deficit-based indices or specific scales such as the TFI. These discrepancies may have influenced the assessment of the interventions’ effectiveness, as clinical outcomes could vary depending on the tools employed. Thus, although all studies evaluated key frailty components such as muscle strength and gait speed, differences in cut-off points and assessment methods hinder direct comparisons of the findings. This review highlights the need for an international consensus on the definition and measurement of frailty, which would enhance comparability between studies and strengthen the body of evidence for future systematic reviews.

On the other hand, this systematic review focused on institutionalised older adults as the study population, specifically to evaluate the effects of physical activity on frailty syndrome. By including only randomised clinical trials, this review ensured a robust methodology suitable for analysing the efficacy of various physical activity interventions, their characteristics, components, and applications in preventing and mitigating frailty in older adults. Randomised trials minimise bias, allow for direct comparisons between intervention and control groups, and provide a realistic representation of how exercise programmes might perform in broader populations following implementation [106].

A noteworthy aspect of this review is the geographical diversity of the included studies, which spanned seven different countries. This diversity highlights the global interest in rigorously investigating the efficacy of physical activity in improving outcomes for older adults, while also enhancing the generalisability of the findings. Moreover, the review specifically focused on frailty, a condition with a higher prevalence among institutionalised older adults due to its multisystem impact. Frailty significantly increases the risk of adverse outcomes, such as disability and mortality [107], underscoring the importance of the findings in this vulnerable population.

## 5. Conclusions

The primary aim of this study was to evaluate the effectiveness of physical activity interventions in preventing and mitigating frailty syndrome among institutionalised older adults. The findings indicate that multicomponent exercise programmes significantly improve frailty parameters, cognitive function, and quality of life, making them a cornerstone in managing this condition.

Multicomponent programs, combining resistance, strength, and aerobic exercises, were found to be safe and effective for addressing various health indicators in frail older adults. These interventions improved functional capacity, including muscle strength, speed, and agility, as well as some biochemical markers such as glucose, total cholesterol, triglycerides, vitamin D3, or CRP. Importantly, they also reduced the number of frailty criteria and reversed frailty in a significant proportion of participants.

This systematic review highlights the malleability of frailty and demonstrates the benefits of interventions across diverse populations, including frail and pre-frail individuals, men and women, and even very old participants in institutional care. The accumulated evidence strongly supports the value of interventions for frailty, particularly those combining physical exercise with nutrition or cognitive training.

While multicomponent exercise programmes show promising results in enhancing overall functional capacity and reducing frailty, the optimal programme design remains unclear. Further research is needed to determine the most effective exercise components, and it should focus on refining specific aspects of interventions, such as training intensity, duration, and frequency, providing stronger evidence for tailored interventions across all levels of care. Additionally, the lack of standardisation in intervention methods poses a critical limitation. Developing internationally recognised guidelines for the design and implementation of frailty interventions would ensure greater consistency and reproducibility, ultimately enhancing their practical applicability and impact on health outcomes in institutionalised older adults.

## Figures and Tables

**Table 1:** Methodological quality assessment of included studies using the PEDro scale.

PEDro Scale												
Study	P1	P2	P3	P4	P5	P6	P7	P8	P9	P10	P11	Total
Ng, et al. [33]	--	Y	N	N	Y	N	Y	Y	Y	Y	Y	**7**
Arrieta, et al. [34]	--	Y	Y	N	Y	N	Y	Y	Y	Y	Y	8
Liu, et al. [53]	--	Y	Y	Y	Y	N	Y	Y	Y	Y	Y	9
Rezola-Pardo, et al. [54]	--	Y	N	N	Y	N	Y	N	Y	Y	Y	6
Courel-Ibáñez et al. [55]	--	Y	Y	Y	N	N	Y	Y	Y	Y	Y	8
Taylor, et al. [56]	--	Y	N	N	N	Y	Y	N	Y	Y	Y	6
Nagaia et al. [57]	--	Y	Y	Y	Y	N	Y	U	Y	Y	Y	8
García-Gollarte et al. [58]	--	Y	Y	Y	Y	N	U	Y	N	Y	Y	7
Batisti-Ferreira et al. [59]	--	Y	Y	N	N	N	Y	Y	Y	Y	Y	7
Tomicki et al. [60]	--	Y	Y	N	N	N	Y	Y	Y	Y	Y	7
López-López et al. [61]	--	Y	Y	Y	Y	N	Y	U	Y	Y	Y	8
Hartantri et al. [62]	--	Y	Y	Y	Y	N	Y	Y	Y	Y	Y	9
%		100	75	50	67	8.3	92	67	92	100	100	

Y: yes; N: no; U: undetermined or unspecified; PEDro scale criteria: 1—Eligibility criteria were specified (this item is not used to calculate the PEDro score); 2—subjects were randomly allocated to groups; 3—allocation was concealed; 4—groups were similar at baseline regarding the most important prognostic indicators; 5—there was blinding of all subjects; 6—there was blinding of all therapists who administered the therapy; 7—there was blinding of all assessors who measured at least one key outcome; 8—measurements of at least one key outcome were obtained from more than 85% of subjects initially allocated to groups; 9—all subjects for whom outcome measures were available received the treatment or control condition as allocated, or when this was not the case, data for at least one key outcome were analysed by “intention to treat”; 10—the results of between-group statistical comparisons were reported for at least one key outcome; 11—the study provided both point measures and measures of variability for at least one key outcome.

**Table 2:** Geographic location, sample characteristics, groups, and functional state of participants (own compilation).

Study	Location	Participants	CG	EG	Age	Functional State
Ng, et al. [33]	Community-dwelling residents in the southwestern region of Singapore	*n* = 98 55 W (56.12%) 43 M (43.88%)	*n* = 50 28 W (56%) 22 M (44%)	*n* = 48 27 W (56.25%) 21 M (43.75%)	>65 yrs GC: 84.7 GI: 85.1	Pre-frail and frail older adults based on Fried et al. [19] criteria, capable of ambulating without personal assistance and without cognitive impairment
Arrieta, et al. [34]	10 nursing homes in Gipuzkoa, Basque Country (Spain).	*n* = 112 79 W (67.05%) 33 M (32.95%)	*n* = 55 37 W (67.3%) 18 M (32.7%)	*n* = 57 42 W (73.7%) 15 M (26.3%)	>70 yrs GC: 84.7 GI: 85.1	Nursing home residents with a Barthel index score of ≥50, capable of standing and walking for at least 10 metres
Liu et al. [53]	8 nursing homes from 3 districts in Harbin, Heilongjiang Province (China)	*n* = 135 40 W (29.63%) 95 M (70.37%)	*n* = 68 24 W (35.29%) 44 M (64.71%)	*n* = 67 16 W (23.88%) 51 M (76.12%)	>75 yrs GC: 80.74 ± 2.82 GI: 80.75 ± 2.99	Older adults meeting one or two points of the frailty phenotype [19], living in nursing homes, without severe chronic or mental illnesses, and capable of walking independently for more than 10 metres
Rezola-Pardo, et al. [54]	9 long-term nursing homes (LTNH) in Gipuzkoa, Basque Country (Spain), distinct from the previous sample	*n* = 85 57 W (67.05%) 28 M (32.94%)	*n* = 43 28 W (65.1%) 15 M (34.9%)	*n* = 42 29 W (69.1%) 13 M (30.9%)	>75 yrs GC: 85.3 GI: 84.9	Nursing home residents with a Barthel index score of ≥50, capable of standing and walking (with or without assistive devices) for at least 10 metres
Courel-Ibáñez et al. [55]	2 nursing homes in Murcia (Spain)	*n* = 24 14 W (58.3%) 10 M (41.7%)	*n* = 12 (EG1)	*n* = 12 (EG2)	>75 yrs EG1: 84.0 ± 10.5 EG2: 87.2 ± 7.6	Institutionalised older adults with sarcopenia, gait speed <0.8 m/s, handgrip strength <26 kg for men and <16 kg for women, and appendicular lean mass adjusted by body mass index <0.789 in men and <0.512 in women
Taylor, et al. [56]	25 long-term care (LTC) facilities in Auckland and Hamilton (New Zealand)	*n* = 520	*n* = 258	*n* = 262	>65 yrs GC: 84.7 GI: 85.1	Institutionalised participants capable of walking and transferring with or without assistance
Nagaia et al. [57]	Older adults living in the community at a rehabilitation centre in Tamba-Sasayama, Hyōgo Prefecture (Japan)	*n* = 41 37 W (90.24%) 4 M (9.76%)	*n* = 20 19 W (95%) 1 M (5%)	*n* = 21 18 W (85.71%) 3 M (14.29%)	>65 yrs GC: 81.8 GI: 81.2	Older adults with at least one frailty criterion from Fried et al. [19], capable of walking independently (or using a cane), and without dementia
García-Gollarte et al. [58]	7 long-term care facilities from the Ballesol Residential Group in Valencia and Alicante (Spain)	*n* = 73 51 W (~70%) 22 M (~30%)	*n* = 34 21 W (61.8%) 13 M (37.2%)	*n* = 39 30 W (76.9%) 9 M (23.1%)	>75 yrs GC: 87.3 GI: 86	Institutionalised older adults without severe cognitive impairment or medical contraindications to performing exercises
Batisti-Ferreira et al. [59]	Long-term care facilities (LTCF) in Brasília (Brazil)	*n* = 37	24 (61.8%)	13 (38.2%)	>60 yrs GC: 77.8 ± 8.0 GI: 73.3 ± 6.4	Pre-frail or frail older adults without limitations that would prevent them from performing cognitive or physical tests
Tomicki et al. [60]	2 philanthropic long-term care centres for older adults located in a municipality in the northern region of Rio Grande do Sul (Brazil)	*n* = 30 19 W (63.3%) 11 M (36.7%)	*n* = 15 9 W (60.0%) 6 M (40%)	*n* = 15 10 W (66.7%) 5 M (33.3%)	>60 yrs GC: 77.3 ± 9.3 GI: 75.1 ± 6.5	Institutionalised older adults diagnosed with frailty, without severe cognitive impairment or degenerative neurological conditions
López-López et al. [61]	Albertia Senior Care Centre in Madrid (Spain)	*n* = 34 25 W (73.5%) 9 M (26.5%)	*n* = 16	*n* = 18	>70 yrs GC: 86.19 GI: 85.78	Nursing home residents with frailty as determined by SPRINTT criteria [18](SPPB ≥3 and ≤9), capable of walking with or without assistive devices, a Barthel index score of ≥50, and the ability to communicate
Hartantri et al. [62]	Jambangan Nursing Home, a government-managed elderly home in Surabaya City (Indonesia)	*n* = 34 18 W (52.94%) 16 M (47.06%)	*n* = 17 9 W (52.9%) 8 M (47.1%)	*n* = 17 9 W (52.9%) 8 M (47.1%)	>60 yrs GC: 77.3 ± 9.3 GI: 75.1 ± 6.5	Older adults in nursing homes with frailty syndrome determined by frailty phenotype criteria [19], capable of ambulating independently, with a Barthel index score of ≥60, and without cognitive impairments

*n*: Number of participants; M: mean; SD: standard deviation; LTNH: long-term nursing homes (care homes); CG: control group; EG: experimental group; M: men; W: women; yrs: years.

**Table 3:** Definition of frailty in included studies with results and assessment tools (own compilation).

Study	Definition of Frailty Used by the Authors	Results and Assessment Tools
Ng et al. [33]	Physical frailty arises from multisystemic physiological decline, increasing risks of hospitalisation, dependency in ADLs, institutionalisation, and mortality	Frailty was assessed using Fried et al.’s criteria [19]. Hospitalisations and falls were self-reported.
Arrieta et al. [34]	Frailty reflects heightened vulnerability to minor stressors, increasing the risk of falls, disability, hospitalisation, institutionalisation, and mortality	Frailty was measured using FFP [19], the SPPB, the SOF index, and the TFI; ADLs were assessed at baseline and after 12 months using the BI [51] which evaluates performance in 10 ADLs.
Liu et al. [53]	A nonspecific condition characterised by a decline in physiological reserve and multisystem dysfunction reducing stress tolerance [64]	Frailty was assessed using changes in the ordinal score of the FFP [19]. Physical gait capacity was evaluated with kinematic parameters (stride length, stride velocity, cadence, and stride time). Cognitive function was measured using the MMSE, and quality of life was assessed with the WHOQOL [65].
Rezola-Pardo et al. [54]	A geriatric syndrome defined by reduced physiological reserve and a consequent increase in vulnerability to stressors	Frailty was evaluated using FFP [19], the SOF index [48] and the TFI [49]. Gait speed was assessed with the short SPPB [66], SFT, and the iTUG. Cognitive function was measured with the MoCA, WAIS-IV symbol coding and search tests, semantic and verbal fluency tests, and the RAVL test. Psychoaffective assessment included the GADS and the JGLS, while perceived quality of life was evaluated with the QLADS [67].
Courel-Ibáñez et al. [55]	Frailty impairs daily activities due to muscle mass and strength loss (sarcopenia and dynapenia), leading to poor health outcomes, reduced functional capacity, fatigue, and falls	Sarcopenia was identified using the FNIH diagnostic algorithm [50]. Functional capacity was assessed with the SPPB, and isometric grip strength was measured using a digital dynamometer.
Taylor et al. [56]	Physical frailty, when combined with cognitive decline, raises the risk of falls and may reduce the adherence to and effectiveness of exercise interventions	Falls were recorded for the six months prior to the trial. Physical capacity was measured using the SPPB [66] and iTUG, while cognitive capacity was evaluated with the MoCA.
Nagaia et al. [57]	Critical syndrome associated with falls, disability, and institutionalisation, leading to premature mortality and high healthcare costs	Frailty was assessed using Fried et al.’s criteria [19]. ADLs were measured using the FAI, a 15-item questionnaire evaluating recent functional activity participation [68]. HRQoL was assessed using the SF-8 health survey.
García-Gollarte et al. [58]	Frailty results in diminished functional performance and is associated with negative health outcomes, making it one of the most significant challenges linked to ageing	Frailty was assessed using Fried et al.’s criteria [19]. Functional balance was measured with the BBS, a 14-item task battery with varying levels of balance difficulty [69].
Batisti-Ferreira et al. [59]	A syndrome characterised by reduced homeostatic reserve and diminished capacity to resist stress, leading to cumulative decline across multiple physiological systems [70]	Functionality was evaluated using the Katz index to measure autonomy in basic ADLs. Depression levels were assessed with the Yesavage scale. Frailty was identified using Fried et al.’s criteria [19].
Tomicki et al. [60]	This study focuses on the risk of falls as a central issue related to frailty	Balance and fall risk were assessed using the TUG test [71] and the BBS.
López-López et al. [61]	The progressive loss of lean mass and subsequent decline in muscle strength associated with ageing are primary causes of sarcopenia	Mobility and balance were measured using the TUG, while gait speed and lower limb function were assessed with the SPPB [66]. ADLs were evaluated at baseline and after 12 months using the BI [51].
Hartantri et al. [62]	Frailty is characterised by fatigue, weight loss, low physical activity, reduced muscle power, and slower gait speed. These frailty phenotypes accumulate, creating a state of vulnerability in older adults	Frailty was determined using FFP [19], and fall risk was assessed with the BBS [69].

FFP: Fried’s frailty phenotype or score; MMSE: mini-mental state examination; WHOQOL: World Health Organization quality of life measurement; SOF index: study of osteoporotic fractures; TFI: Tilburg frailty indicator; SPPB: short physical performance battery; SFT: senior fitness test; TUG: timed up-and-go test; iTUG: instrumented timed up-and-go; MoCA: Montreal cognitive assessment; WAIS-IV: Wechsler adult intelligence scale; RAVLT: Rey’s auditory verbal learning test; GADS: Goldberg anxiety and depression scale; JGLS: Jong Gierveld loneliness scale; QoL-AD: quality of life in Alzheimer’s disease scale; ADLs: activities of daily living; BI: Barthel index; FNIH: Foundation for the National Institutes of Health; FAI: Frenchay activities index; BBS: Berg balance scale; HRQoL: health-related quality of life; SF-8: short-form 8-item health survey.

**Table 4:** Fried phenotype (own compilation based on Fried et al. [19]).

**Unintentional Weight Loss** **Sarcopenia (Loss of Muscle Mass)**	>10 pounds (4.5 kg) lost involuntarily in the past year	1 point
**Weakness**	Grip Strength: 20% lower (adjusted for gender and body mass index)	1 point
**Slow Walking Speed**	Time for 15 Steps: 20% slower (adjusted for gender and height)	1 point
**Low Physical Activity**	Kcal/week: 20% lower; men: <383 Kcal/week, women: <270 Kcal/week.	1 point
**Low Endurance; Exhaustion**	Self-Reported Exhaustion: Positive if experienced >3–4 days per week or most of the time.	1 point

Each variable is scored with one point. A diagnosis of frailty requires the presence of three or more positive criteria. The presence of one or two criteria indicates an intermediate diagnosis of pre-frailty.

**Table 5:** Interventions documented in the studies included in this review, adverse effects, and dropouts related to the interventions (own compilation).

Study	EG	CG	Length of the Intervention	Adverse Effects	Dropouts
Ng et al. [33]	Strength and balance training following ACSM guidelines with functional and resistance exercises at 60–80% of 10 RM [72]. This study also included experimental groups not related to physical activity directly: a nutritional group, cognitive training group, and combinative interventional group with all three programmes.	Usual community care services, including a placebo nutritional intervention.	Sessions were 90 min long, conducted twice weekly for 12 w, led by a qualified trainer, followed by 12 w of home exercises	Joint pain in two patients solved by load adjustment	- Low dropout rate. - Reasons: medical diagnoses (tuberculosis, lymphoma); change of residence; deaths (2 cases).
Arrieta et al. [34]	Progressive multicomponent interventions incorporating strength, flexion, adduction, and balance exercises. Intensity was gradually increased from 40% at the beginning to 70% of 1RM by the sixth month of the programme.	Low-intensity routines typically offered in care homes, such as memory workshops, reading, singing, and light gymnastics.	EG attending supervised group training sessions of 1 h twice weekly for 6 months	No adverse effects reported	- Attendance rate: 90.8%. - No significant dropouts occurred during the study.
Liu et al. [53]	Integrated exercise intervention plans included Tai Chi and exercises like chest extensions, trunk extensions, walking, squats, and knee extensions [73].	Participants engaged in usual activities.	The intervention group participated in a 40 min sessions five times per week for 12 months, while the control group received no intervention during this period	No adverse effects reported	- Dropout rate: 7.53%. - Reasons: 2 participants did not meet the inclusion criteria; 4 refused to participate in the follow-up assessment; 5 died during the study.
Rezola-Pardo et al. [54]	Individually tailored multicomponent strength and balance exercises performed at moderate intensity.	Dual-task training programme combining cognitive training and the same exercises conducted by the multicomponent group.	3 months	No adverse effects reported	- Adherence rate: 91.4% in the multicomponent group; 84.8% in the dual-task group. - No specific reasons for dropping out were mentioned.
Courel-Ibáñez et al. [55]	Personalised Vivifrail multicomponent exercise programme which prescribes individualised exercise based on the functional capacity of older adults (disability, frailty, pre-frailty, and robustness). Regimens included resistance/power training, balance, flexibility, and cardiovascular endurance exercises.		LT-SD: 24 w of training followed by 6 w of detraining; (ST-LD): 4 w of training followed by 14 w of detraining	No adverse effects reported	- Dropout rate: 8.4%. - Reasons: 2 dropouts in the long training group (LT-SD) due to loss of interest.
Taylor et al. [56]	Staying UpRight (S-UpR) programme with progressive balance and strength exercises, increasing task complexity.	Group seated activities without resistance or progression, such as seated swimming, walking, and stretching.	S-UpR classes were delivered twice weekly for 1 h over 12 months	No serious adverse effects were reported, only one fall without injury in the training process	- Reasons for abandonment: deaths; changes in mobility; interruptions related to the COVID-19 pandemic.
Nagaia et al. [57]	Resistance exercises involved leg presses, knee extensions, leg abductions, and seated rows. Exercise intensity progressively increased from 50% to 80% of 1 RM [74], with 1–2 min rest intervals between sets. In addition to this RT, participants were motivated and instructed to increase physical activity and step count, while reducing sedentary time by 10% every 14 days. Goal setting information and feedback were also provided.	RT training twice weekly as the EG in the same conditions.	24 w period	No adverse effects reported	- Dropout rate: 22%. - Reasons: hospitalisations due to chronic illness (5 participants); interruption of the use of facilities for personal reasons (4 participants).
García-Gollarte et al. [58]	The OEP included sessions focusing on balance, strength, and aerobic exercises, complemented with walking at the end. Participants used elastic bands as external resistance for strengthening exercises. The OEP+N followed the same exercise protocol as the OEP group but included a nutritional supplement of ENSURE (35 g) taken twice daily, designed to preserve muscle mass in older adults.	Participants did not receive any intervention and were asked to continue with their usual daily activities.	A total of 72 sessions were conducted over a 24 w intervention (6 months), with each of the 4 programme levels lasting 6 w	The OEP+N group presented cases of intolerance to the nutritional supplement	- Dropout rate: OEP+N: 44.7%; OEP: 23.1%. - Reasons for dropout in OEP+N: intolerance to nutritional supplement.
Batisti-Ferreira et al. [59]	Multicomponent exercises focused on improving mobility, flexibility, strength, and aerobic endurance.	Participants received no intervention and maintained their usual daily activities.	Sessions were conducted three times per week for 12 w, with each session lasting 40 min	No adverse effects reported	- Reasons: change of institution; refusal to continue in the study; physical limitations that prevented the initial assessment.
Tomicki et al. [60]	The exercise programme included a warm-up (8–10 min), a main session (15–20 min) with aerobic resistance, strength and muscular endurance, flexibility, static and dynamic balance, agility, and motor coordination exercises, followed by stretching and relaxation (8–10 min).	No intervention was provided; participants only engaged in the routine activities offered by the institution.	The intervention lasted 12 w, with sessions held three times weekly on alternate days, totalling 36 sessions, each lasting approximately 45 min	No adverse effects reported	No dropouts reported.
López-López et al. [61]	A multicomponent training programme began with a 5 min activation period, including walking at a normal speed (measured during SPPB evaluation) on a treadmill. Participants then performed two resistance exercises to enhance lower limb muscle power and plantar flexion using step exercises, followed by an aerobic and interval treadmill protocol.	A residential care exercise programme focused on active mobility for most joint groups of the limbs.	Over 12 w, a total of 32 sessions were conducted at a rate of 2 sessions per week, each lasting about 45 min, with at least 48 h between sessions	No adverse effects reported	No dropouts reported.
Hartantri et al. [62]	The Vivifrail multicomponent exercise programme was designed for older adults with varying functional capacities and fall risks. It incorporated upper and lower limb strengthening exercises, flexibility and balance training, and cardiorespiratory endurance exercises.	Routine morning activities were organised for all nursing home residents, including a programme of low-intensity aerobic and stretching exercises lasting 10 to 15 min daily.	4 w period	No adverse effects reported	2 dropouts (1 in the intervention group and 1 in the control group) due to moving to live with relatives.

EG: experimental group; CG: control group; RM: repetition maximum; S-UpR: Staying UpRight; ACSM: American College of Sports Medicine; LT-SD: long training, short detraining; ST-LD: short training, long detraining; RT: resistance training; min: minutes; h: hours; w: weeks; OEP: Otago exercise programme; OEP+N: Otago exercise programme with nutritional supplementation; ENSURE: nutritional supplement by Abbott Laboratories, Indianapolis, Indiana; SPPB: short physical performance battery.

**Table 6:** Analysis of interventions in the included studies (own compilation).

Study	Intervention	Results	Interpretation
Ng et al. [33]	Strength and Balance Training	Frailty score EG Initial value: 2.2 (0.85) At 6 months: 1.3 (0.87) At 12 months: 1.4 (0.80) Frailty reduction, *n* (%) at 12 months: 19 (41.3) CG Initial value: 1.8 (0.80) At 6 months: 1.4 (1.06) At 12 months: 1.6 (0.97) Frailty reduction, *n* (%) at 12 months: 7 (15.2)	There was a significant main effect of time (*p* < 0.001), with a reduction in the mean frailty score over 12 months across all groups, and a significant interaction between group and time (*p* < 0.044). At 12 months, all interventions demonstrated significant differences compared to the control group at the pre hoc significance level of *p* < 0.05.
Arrieta et al. [34]	Multicomponent Exercise	FFP (0–5 range) Initial value CG: 2.8 ± 1.1; EG: 2.8 ± 0.9 At 6 months CG: 3.0 ± 1.2; EG: 2.6 ± 0.9 SPPB (0–12 range) Initial value CG: 5.8 ± 2.7; EG: 6.1 ± 3.1 At 6 months CG: 4.9 ± 2.8; EG: 7.9 ± 3.1 TFI (0–15 range) Initial value CG: 5.9 ± 2.7; EG: 5.8 ± 3.0 At 6 months CG: 5.4 ± 3.1; EG: 4.3 ± 2.9	FFP, SPPB, SOF index, and TFI showed no significant differences in frailty rates between the CG and EG before the intervention (*p* > 0.05). However, after the 6-month intervention, frailty prevalence was significantly lower in the EG compared to the CG, as measured by FFP (53.7% *vs.* 75.8%; *p* < 0.05), SPPB (67.4% vs. 93.0%; *p* < 0.05), and TFI (41.9% vs. 65.7%; *p* < 0.05). SPPB scores significantly decreased in the CG and improved in the IG after 6 months (*p* < 0.05).
Liu et al. [53]	Integrated Exercise Intervention Plan	FFP Pre-intervention CG: 2.71 ± 0.79 EG: 2.51 ± 1.01; *p* = 0.205 Post-intervention CG: 2.68 ± 0.84 EG: 1.07 ± 1.32; *p* < 0.001	After 12 months of intervention, FFP significantly decreased in the EG (*p* < 0.001) but not in the CG. Post-intervention, the FFP in the EG was significantly lower than in the CG (t = 8.445, *p* < 0.001). Additionally, the mean stride velocity, step length, and cadence showed significant improvements (all *p* < 0.001) in the EG compared to the CG after the intervention.
Rezola-Pardo et al. [54]	Multicomponent Exercise and Dual-Task Training	Physical performance (SPPB) Multicomponent Group (*n* = 33) Initial value: 6.8 (3.1) At 3 months: 8.3 (3.1) Dual-Task Group (*n* = 35) Initial value: 7.1 (2.9) At 3 months: 8.7 (2.9) 6-minute walk test Multicomponent Group (*n* = 33) Initial value: 267 (118) At 3 months: 284 (112) Dual-Task Group (*n* = 35) Initial value: 282 (105) At 3 months: 293 (110)	Both groups showed significant improvements in physical performance parameters, with an increase of ~1.6 points in the SPPB test for both groups (*p* < 0.001). However, only the multicomponent group demonstrated a significantly improved performance in the 6-minute walk test and TUG tests post-intervention (*p* < 0.05).
Courel-Ibáñez et al. [55]	Vivifrail Multicomponent Exercise Programme	SPPB Adjusted mean (IC95%) LT-SD 8.7 (7.3; 10.2) ST-LD 6.6 (5.3; 8.0) *p =* 0.035 TUG test LT-SD 18.4 (14.7; 22.1) ST-LD 19.4 (16.2; 22.5) *p >* 0.05	Both groups responded positively to the 4-week Vivifrail multicomponent training programme, significantly improving their functional and strength parameters (effect size [ES] 0.32 to 1.44; *p* < 0.05), except for handgrip strength in the LT-SD group. Additional training in the LT-SD group over the following 20 weeks resulted in significant improvements across all variables (ES 0.80 to 1.51), except for handgrip strength.
Taylor et al. [56]	S-UpR Programme	Falls Initial value CG: 3.3; EG: 4.1 ppy At 6 months CG: 4.3; EG: 4.1 ppy Walking speed (m/s) Initial value CG: 0.61 (0.4); EG: 0.61 (0.4) At 6 months CG: −0.1 (0.6); EG: −0.1 (0.7) SPPB (0–12 range) Initial value CG: 4.8 (2.9); EG: 4.6 (2.6) At 6 months CG: −0.4 (1.4); EG: −0.3 (1.4)	SPPB scores decreased by 1.3% over a 10-month period (0.6 points, 95% CI: 0.3, 0.8), with no significant differences between the EG and CG. Step count declined by 3% over the same period (544 steps/day, 95% CI: 181, 908), also showing no significant differences between groups. However, compared to the control group, step count was better preserved in the EG participants who had higher adherence (*n* = 24) (≥48 classes) (*p* = 0.020).
Nagaia et al. [57]	Resistance Exercises (RT) Resistance Training with Physical Activity (RPA)	TUG test RT RPA Pre: 12.6 (4.6) 12.8 (3.7) Post: 12.4 (5.9) 11.1 (2.8) Walking Speed (m/s) RT RPA Pre: 0.81 (0.21) 0.76 (0.22) Post: 0.85 (0.20) 0.84 (0.21)	Post-intervention frailty status did not differ significantly between groups (*p* = 0.636, Cramer’s V = 0.029). However, frailty scores in the RPA group significantly decreased following the intervention (group × time interaction: *p* = 0.023, F = 5.632, η^2^ = 0.126). Significant main effects of time were observed for walking speed and TUG scores, representing mobility (*p* < 0.05); however, the group × time interaction effect was not significant.
García-Gollarte et al. [58]	Otago Exercise Programme (OEP) Otago Exercise Programme with Nutritional Supplementation (OEP+N)	TUG test OEP OEP+N Pre: 27.6 (18.3) 20.6 (17.5) Post: 16.2 (1.5) 23.5 (1.4) GC Pre: 21 (9.5) Post: 24.4 (1.5) BBS (0–56 range) OEP OEP+N Pre: 36.7 (12.6) 38.9 (10.7) Post: 42.4 (0.8) 38.8 (0.9) GC Pre: 38.7 (9.6) Post: 34.2 (0.9)	After the intervention, the OEP group demonstrated significant improvements in the TUG test compared to their own pre-intervention values and to the other groups (*p* < 0.001). In terms of handgrip strength (HG), both the OEP and OEP+N groups showed significant improvements compared to the control group (*p* < 0.001). However, only the OEP group exhibited a positive change between pre and post (14.6 vs. 16.1; 0.7 [−0.3 to 1.8]), although it was not statistically significant. The OEP and OEP+N groups showed an 8.2- and 4.6-point improvement for BBS scores compared to the CG (*p* < 0.001), as well as the OEP group achieving significantly higher BBS scores than the OEP+N (3.5 points, *p* = 0.011).
Batisti-Ferreira et al. [59]	Multiple-Component Exercises	Left-hand grip strength EG CG Pre: 8.7 16.7 Post: 16.7 10.5 TUG test EG CG Pre: 28.8 29.1 Post: 20.9 28.9	Pre–post-intervention functional performance variables significantly improved in the EG. The EG demonstrated 33% and 26% higher left- and right-hand grip strength vs. CG. Additionally, EG showed significantly lower scores in the TUG and sit-to-stand tests (38% and 29% lower, respectively) vs. CG.
Tomicki et al. [60]	Multiple-Component Exercises	TUG test EG CG Pre: 17.0 17.0 Post: 9.0 19.0 BBS EG CG Pre: 49 49 Post: 52 46	Regarding the tests and fall frequency, there was a significant correlation between baseline TUG and BBS scores (rs = −0.80, *p* < 0.001). The results indicate that older adults who engaged in regular physical exercise for three months did not experience any falls in contrast to the CG participants.
López-López et al. [61]	Multicomponent Training Programme	Barthel index EG CG Pre: 74.17 73.75 Post: 67.78 72.81 10-minute walk test EG CG Pre: 10.20 8.53 Post: 7.79 9.76 SPPB EG CG Pre: 5.33 5.44 Post: 6.94 4.31	The CG increased their TUG test times, while the EG reduced theirs after training, showing an improvement of 7.43 s compared to the CG (95% CI: 3.28–11.59). The EG reduced their time by 5.19 s compared to the CG after training (95% CI: 1.41–8.97), improving their SPPB score as well.
Hartantri et al. [62]	Vivifrail Multicomponent Exercise Programme	BBS Vivifrail CG Pre: 48.59 ± 5.45 46.47 ± 5.57 Post: 52.65 ± 3.66 45.29 ± 7.43 *p* = 0.001 *p =* 0.298 *p =* 0.001 (within groups) FES-I Vivifrail CG Pre: 22.76 ± 5.04 23.06 ± 7.26 Post: 20.06 ± 4.63 26.24 ± 9.93 *p* = 0.025 *p =* 0.096 *p =* 0.005 (within groups)	After 4 weeks of exercise, the mean BBS in the EG increased to 52.65 (*p* < 0.01), while the CG decreased to 45.29 but not significantly. Between-group analysis revealed a statistically significant improvement in the EG with a very large effect size (1.33). Only the EG improved the FES score, being quite better compared to the CG, who increased the score after the intervention period.

*n*: number of participants; CG: control group; EG: experimental group; Pre: values before the intervention; Post: values after the intervention; CI: confidence interval; LT-SD: long training (24 w), short detraining (6 w); ST-LD: short training (4 w), long detraining (14 w); S-UpR: Staying UpRight; FFP: Fried’s frailty phenotype or score; TUG: timed up-and-go test; SOF index: study of osteoporotic fractures; TFI: Tilburg frailty indicator; SPPB: short physical performance battery; ppy: per person-year; BBS: Berg balance scale; HG: handgrip; FES-I: fall efficacy scale.

## Data Availability

The raw data supporting the conclusions of this article will be made available by the corresponding or last authors of the manuscript on request.
